# Supplementary material for: Graphitized-rGO/Polyimide Aerogel as the Compressible Thermal Interface Material with Both High in-Plane and through-Plane Thermal Conductivities
Source: Materials (Basel). 2021 Apr 30;14(9):2350. doi: 10.3390/ma14092350 (PMC8125293; doi:10.3390/ma14092350)
Supplement: Supplementary file 1 [file materials-14-02350-s001.zip › materials-1175590-supplementary.pdf]

materials

MDPI

Article

Graphitized-rGO/Polyimide Aerogel as the Compressible Thermal Interface Material with Both High In-Plane and Through-Plane Thermal Conductivities

Peng Lv *, Haiquan Cheng, Chenglong Ji and Wei Wei

Department of Optoelectronic Information Science and Engineering, College of Electronic and Opitical Engineering, Nanjing University of Posts&Telecommunications, Nanjing 210023 China; 1218022710@njupt.edu.cn (H.C.); jichenglong2021@163.com (C.J.); weiwei@njupt.edu.cn (W.W.)
* Correspondence: lvp@njupt.edu.cn; Tel.: +86-25-8586-6296

Supporting information

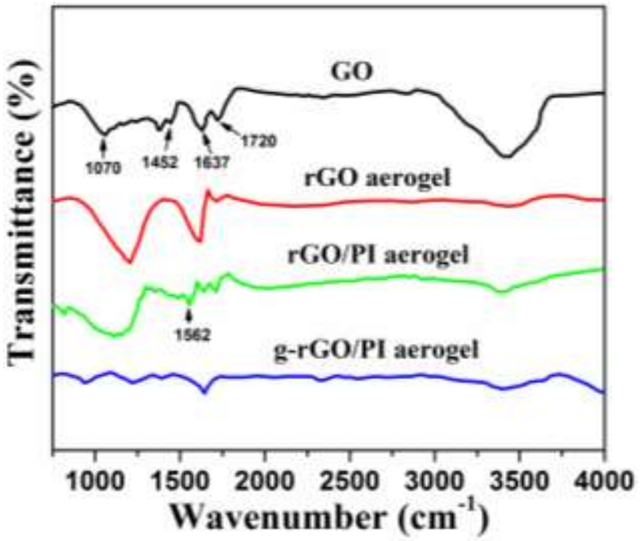

Figure 1. FTIR spectra of GO, rGO aerogel, rGO/PI aerogel and g-rGO/PI aerogel.

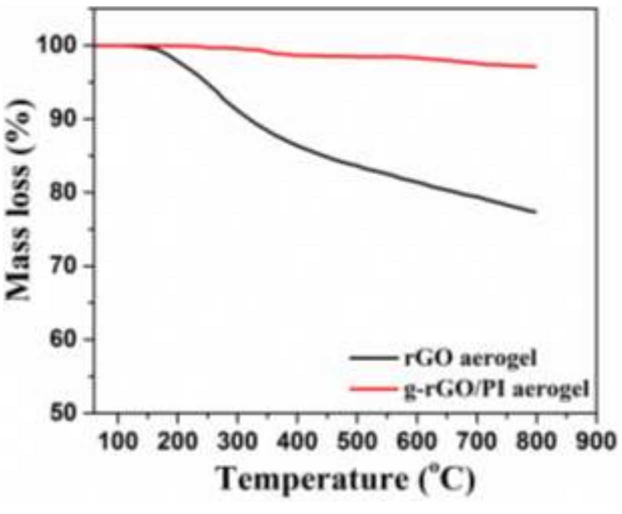

Figure 2. TGA curves of rGO aerogel and g-rGO/PI aerogel.

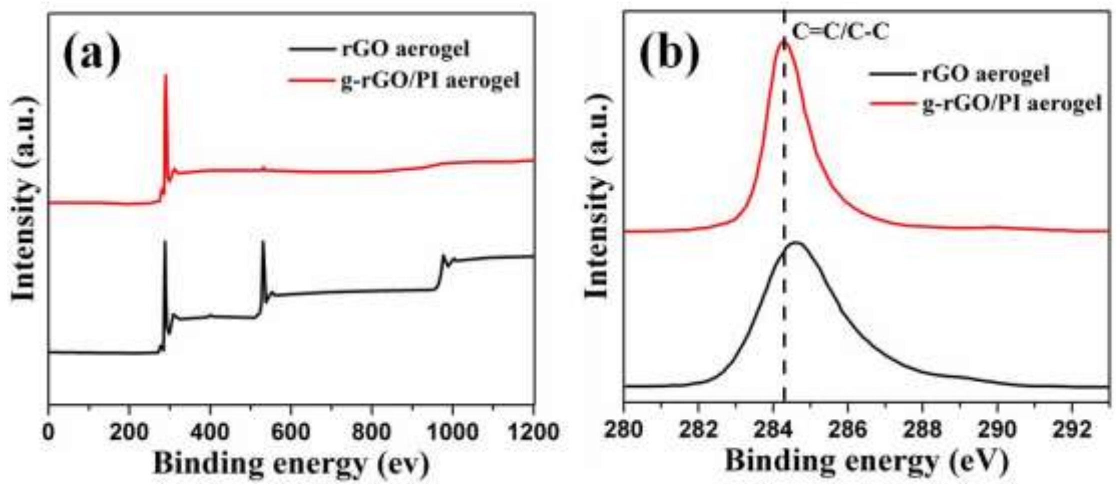

Figure 3. (a) XPS spectra of the elemental composition of rGO aerogel and g-rGO/PI aerogel; (b) High-resolution XPS analysis (C1s).
